# Supplementary material for: PPM1D mutations are oncogenic drivers of de novo diffuse midline glioma formation
Source: Nat Commun. 2022 Feb 1;13:604. doi: 10.1038/s41467-022-28198-8 (PMC8807747; doi:10.1038/s41467-022-28198-8)
Supplement: Supplementary file 1 — Supplementary Information [file 41467_2022_28198_MOESM1_ESM.pdf]

Supplementary Information

Supplementary Note 1: Landscape of PPM1D alterations in human cancers
Our analysis of WGS data of 170 pHGGs confirmed previous findings (Mackay et al., 2017; Taylor et al., 2014; Wu et al., 2014; Zhang et al., 2014) that PPM1D is recurrently mutated in these cancers (Supplementary Figure 10A). Two analyses indicated that PPM1D mutations tend to be clonal events in our cohort of gliomas. First, PPM1D mutations were among the mutations with the highest variant allele fractions (VAFs) within individual tumors (mean VAF = 0.37 +/- 0.17) (Supplementary Figure 10B). However, VAF can be influenced by tumor purity and ploidy. Indeed, integration of these VAF data with copy-number data (controlling for both purity and ploidy) from these gliomas using the ABSOLUTE algorithm (Carter et al., 2012) indicated a median cancer cell fraction (CCF) of 1, suggesting clonality (i.e. the mutation was present in all tumor cells) (Supplementary Figure 10C). Second, whole genome sequencing of two PPM1D-mutant DMGs for which paired pre and post treatment samples were available revealed both biopsy and autopsy samples to harbor the same PPM1D mutation with CCFs of 1 (Supplementary Data 12). This included one PPM1D-mutant DMG for which we had a biopsy obtained at diagnosis and six matched multiregional samples obtained at autopsy from the same patient revealed the same PPM1D mutations in all samples (mean VAF = 0.64 +/- 0.03, median CCF = 1) (Supplementary Figure 10D-E). Taking these analyses together, we conclude that PPM1D mutations are early events in gliomagenesis.

In addition to truncating PPM1D mutations, analysis of our copy-number data of pHGGs revealed broad low-level gains in the PPM1D containing region on 17q, but no focal amplification of PPM1D was detected (Supplementary Figure 11A). Moreover, we observed a significant correlation between PPM1D copy-number and expression level in a cohort of 116 pHGGs, including 9 PPM1D mutant tumors ($R = 0.41$, $P < 5.6e-06$) (Supplementary Figure 11B). PPM1D expression was also significantly higher in PPM1D-mutant gliomas compared to the wild-type ones (Supplementary Figure 11C), but no difference was observed between DMGs and hemispheric gliomas (Supplementary Figure 11D). Extending our copy-number analysis to other cancers in the TCGA dataset, we observed PPM1D amplifications in 0.3% of 6568 pediatric tumors. Among adult tumors, PPM1D amplifications were present in 2% of 10,967 adult cancers. These amplifications were statistically recurrent in several cancer types including bladder urothelial carcinoma (q-value = 0.0452), breast adenocarcinoma (q-value = 3.93 x 10-5), epithelial cancers (q-value = 2.04 x 10-31), liver hepatocellular carcinoma (q-value = 2.68 x 10-4), colorectal cancers (q-value = 0.0111), colon adenocarcinoma (q-value = 0.0704) and melanomas (q-value = 0.244). However, PPM1D was located in the consensus amplification peak predicted to contain oncogenic driver gene only in bladder urothelial carcinoma but not in any of the other cancer types.

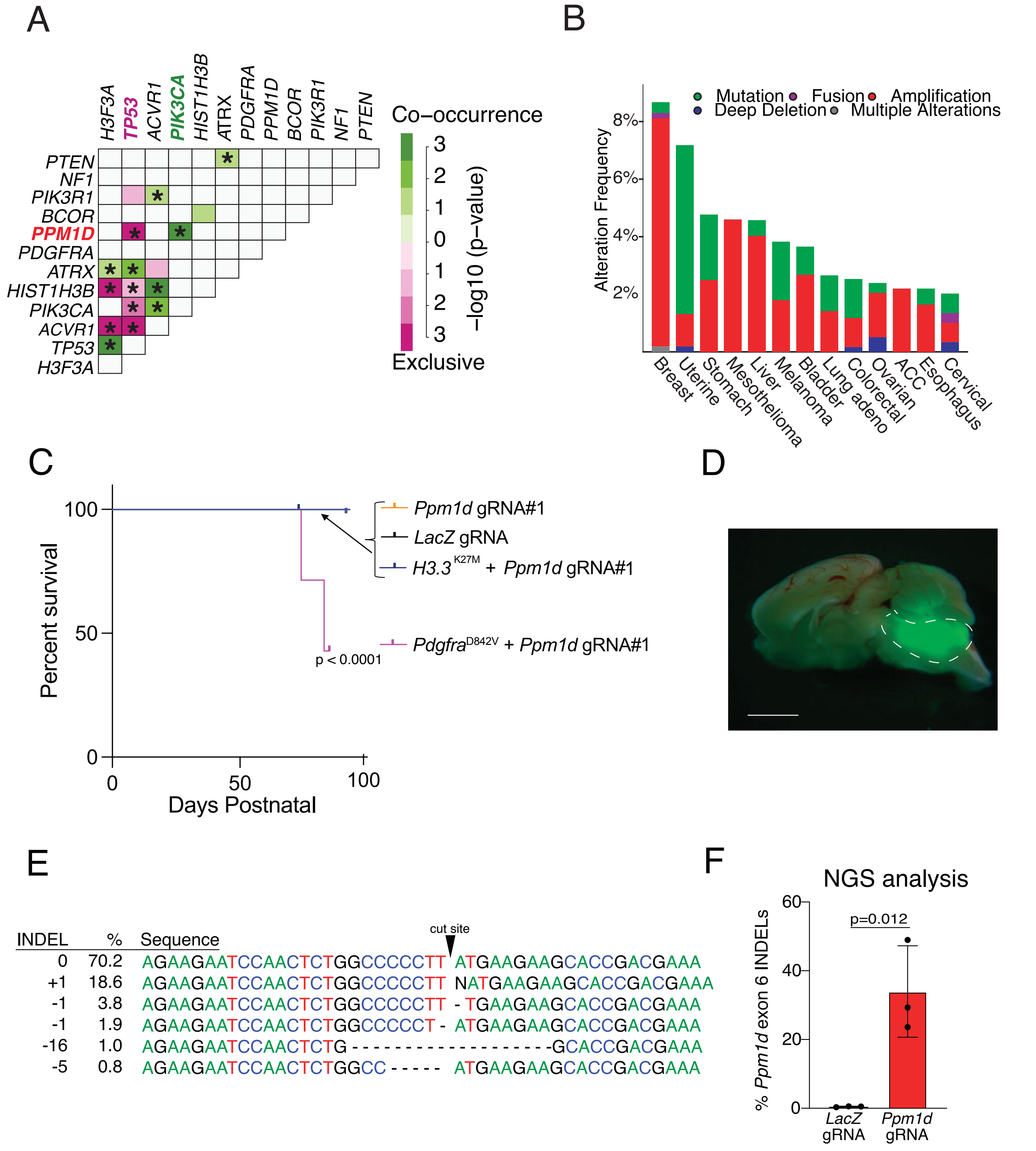

Supplementary Figure 1. A) Pairwise association of genes co-occurring and mutually exclusive with PPM1D mutations performed using the Fisher's exact test. Green depicts co-occurrence while red depicts mutual exclusivity. *Depicts P-value < 0.05 calculated using Fisher's exact test as computed by Maftools package. B) Alterations in PPM1D in various human cancers from the TCGA pan-cancer study. C) Kaplan Meier survival curve for single gene IUE conditions: Ppm1d gRNA#1 (n=6), LacZ gRNA (n=9), PdgfraD842V + Ppm1d gRNA#1 (n=7), H3.3K27M + Ppm1d gRNA#1 (n=7). P < 0.0001 for LacZ gRNA vs PdgfraD842V + Ppm1d gRNA#1 condition determined using log rank test. D) Midline image of a GFP-positive Ppm1d gRNA IUE DMG mouse model. Circle denotes GFP-positive tumor region. Similar imagings were performed in a minimum of three independent samples. Scale bar denotes 2.5mm. E) INDEL sequences and rates (%) identified by TIDE analysis of Ppm1d exon 6 sequence in Ppm1d gRNA IUE DMG tumor. F) Percentage of modified NGS reads in Ppm1d gRNA IUE DMG tumors (n=3) compared to LacZ gRNA condition (n=3). P = 0.0121 calculated using two-tailed t-test. Data presented as mean +/- S.E.M of three biological replicates. Source data are provided as a Source Data file.

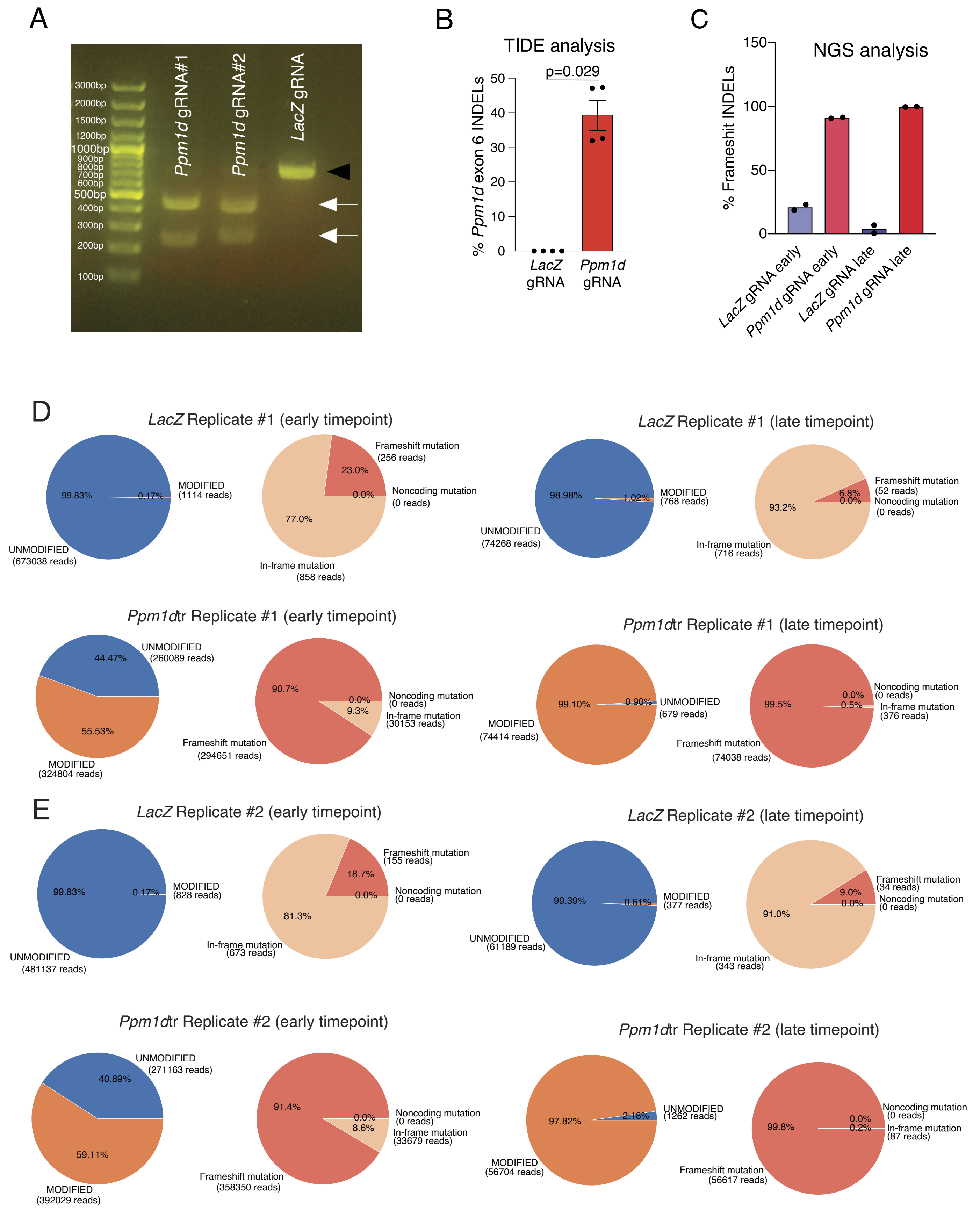

Supplementary Figure 2. A) Cell-free in vitro assay of Ppm1d gRNA#1, Ppm1d gRNA#2 and LacZ gRNAs directed cutting of the Ppm1d exon6 locus by Cas9. Agarose gel shows the ~800bp PCR product of Ppm1d exon6 is efficiently cut by sgRNA/Cas9 conditions targeting Ppm1d, but not LacZ. Similar results were obtained in two independent experiments. B) Depiction and quantification of INDEL sequences and rates identified by TIDE analysis of Ppm1d exon6 using gDNA from mouse embryonic neural stem cells electroporated with CRISPR-Cas9 plasmids encoding Ppm1d gRNA#1 or LacZ gRNA. Bar graph of Ppm1d exon6 INDEL rate across experiments (n=4 per condition). P = 0.029 for LacZ gRNA vs Ppm1d gRNA#1 condition calculated using two-tailed Mann-Whitney test. Data presented as mean +/- S.E.M. C) Percentage of modified NGS reads with Ppm1d frameshift INDELs in cells with guides against LacZ or Ppm1d exon 6 at an early time point compared to late time point. Data shows mean of two independent replicates. D-E) Pie charts showing quantification of modified and unmodified reads as well as frameshift and in-frame NGS reads at both early and late time points in cells infected with guides against LacZ or Ppm1d exon 6. Source data are provided as a Source Data file.

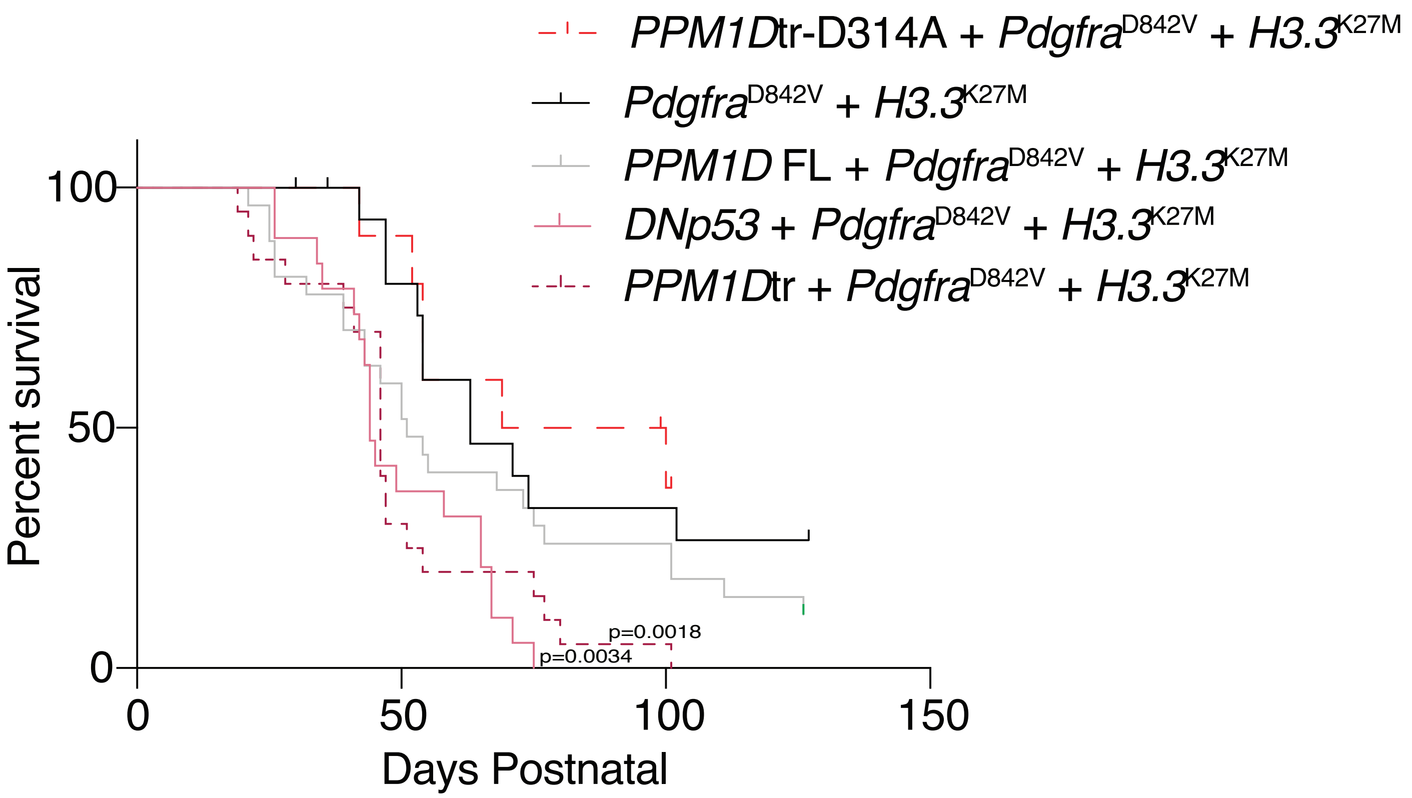

Supplementary Figure 3. Kaplan Meier survival curves for DNp53 (n=19) and PPM1D FL (n=27) IUE DMG mouse models along with control arms (n=19), PPM1Dtr (n=20), and PPM1Dtr-D314A (n=10) for the data presented in Figure 2C. P = 0.0018 and 0.0034 for control vs PPM1Dtr and control vs DNp53 conditions respectively calculated using log-rank Mantel-Cox test. Source data are provided as a Source Data file.

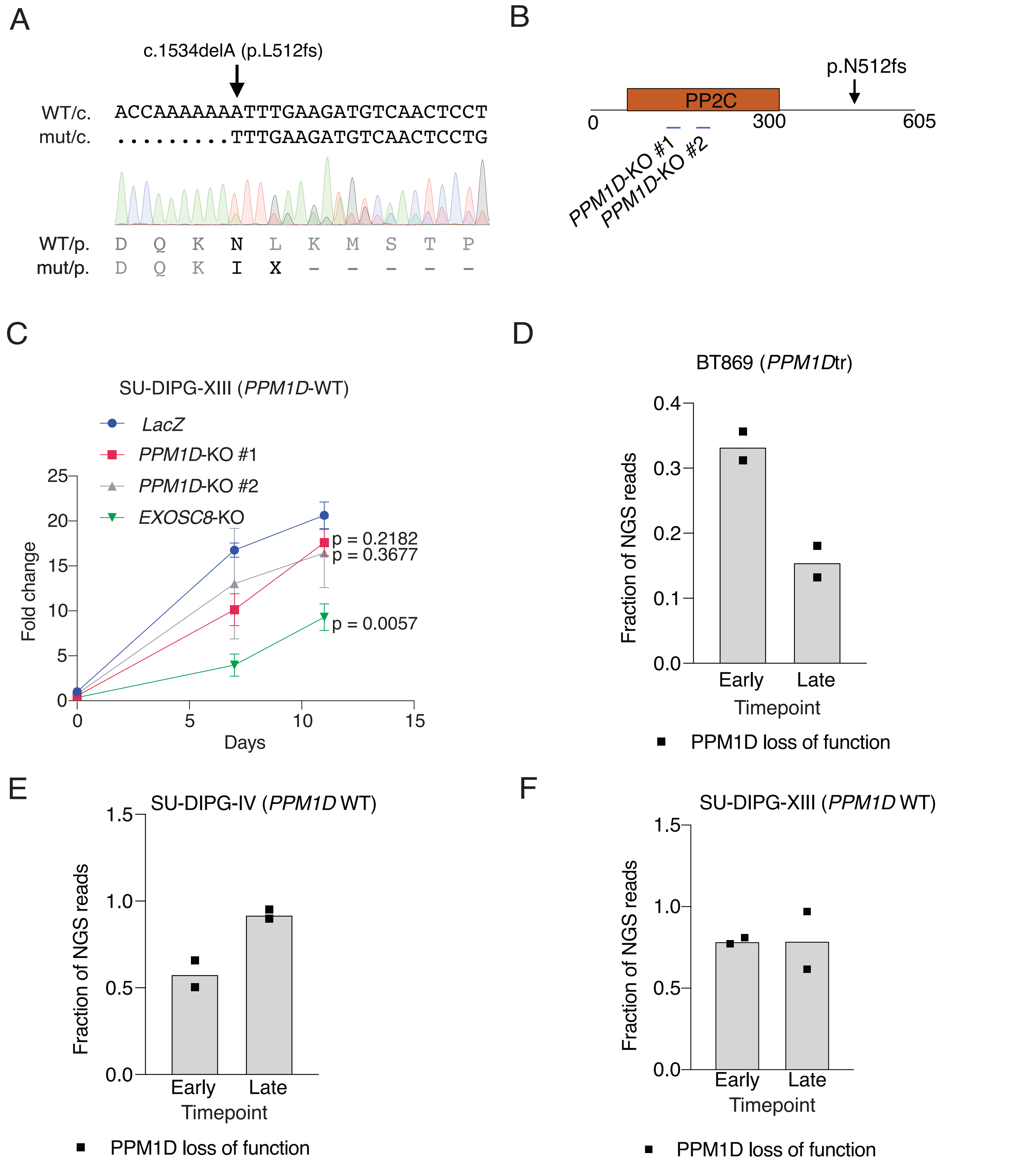

Supplementary Figure 4. A) Sanger sequencing across PPM1D in the BT869 DMG cell line reveals a truncating PPM1D mutation (p.N512fs). B) Schematic shows locations of PPM1D-targeting guides within the PPM1D gene. The PP2C phosphatase domain and N512fs mutations are shown. C) Growth of PPM1D-WT PDCL SU-DIPG-XIII after transfection with PPM1D-KO sgRNAs, nontargeting (LacZ) sgRNA or lethal (EXOSC8) sgRNA. Growth curves show mean +/- S.E.M. from three replicates and are representative of three independent experiments. P = 0.2182, 0.3677, and 0.0057 for PPM1D-KO #1, PPM1D-KO #2, and EXOSC8 respectively calculated using two-tailed t-test. D-F) Next generation sequencing assays to determine the proportion of sequencing reads that harbor loss-of-function PPM1D edits at exon 2. Cell lines were sequenced immediately after selection following Cas9 transduction and sgRNA transduction (early), and then after ten cell doublings (late). NGS results for BT869 (D), SU-DIPG-IV (E), and SU-DIPG-XIII (F) are shown. Results show mean from two independent experiments carried out with two independent PPM1D-KO sgRNAs. Source data are provided as a Source Data file.

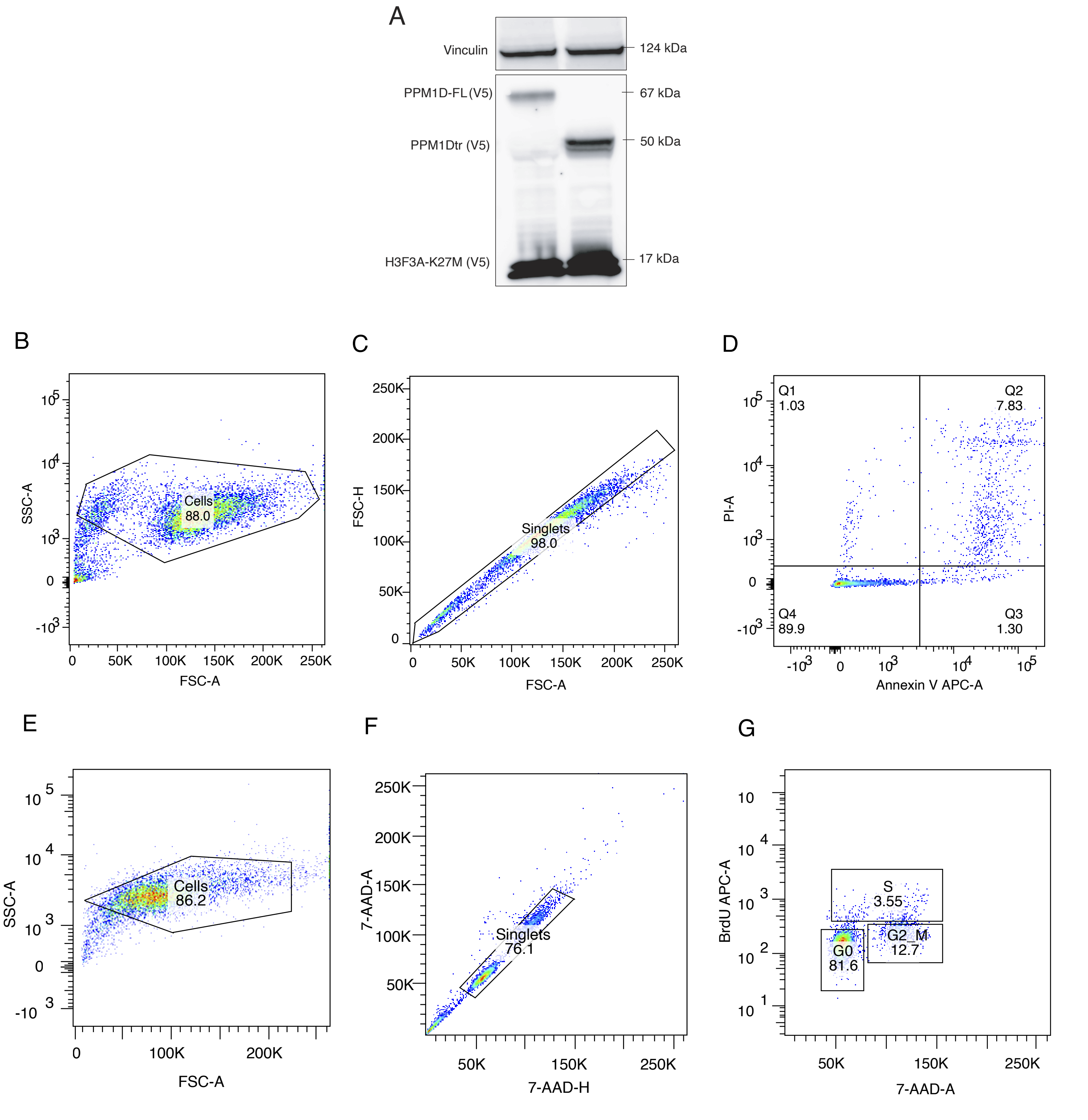

Supplementary Figure 5. Cells were sequentially infected with vectors overexpressing V5-tagged H3F3A K27M and either PPM1D FL or PPM1Dtr. Lysates were collected after the lines were established, subjected to SDS-PAGE, and probed with the indicated antibodies (see methods for antibodies used). B-G) Example of flow cytometry gating schemes for apoptosis (B-D) and cell-cycle (E-G) experiments performed on mNSC overexpressing GFP (negative control). B) Forward scatter area (FSC-A) and side scatter area (SSC-A) plot of cells with gating on cells for the apoptosis experiment. C) Further gating of cells to identify singlets using forward scatter area and height. D) Identification of various populations of cells using Annexin V APC area (Annexin V APC-A) and PI area (PI-A). Q1 is necrotic population, Q2 is late-apoptotic population, Q3 is early-apoptotic population, and Q4 is live population. E) Forward scatter area (FSC-A) and side scatter area (SSC-A) plot of cells with gating on cells for the cell-cycle experiment. F) Further gating of cells to identify singlets using 7-AAD area (7-AAD-A) and height (7-AAD-H). G) Identification of populations of cells in different stages of cell cycle (G0, S, and G2-M) as indicated using 7-AAD area (7-AAD-A) and BrdU-APC area (BrdU-APC-A).

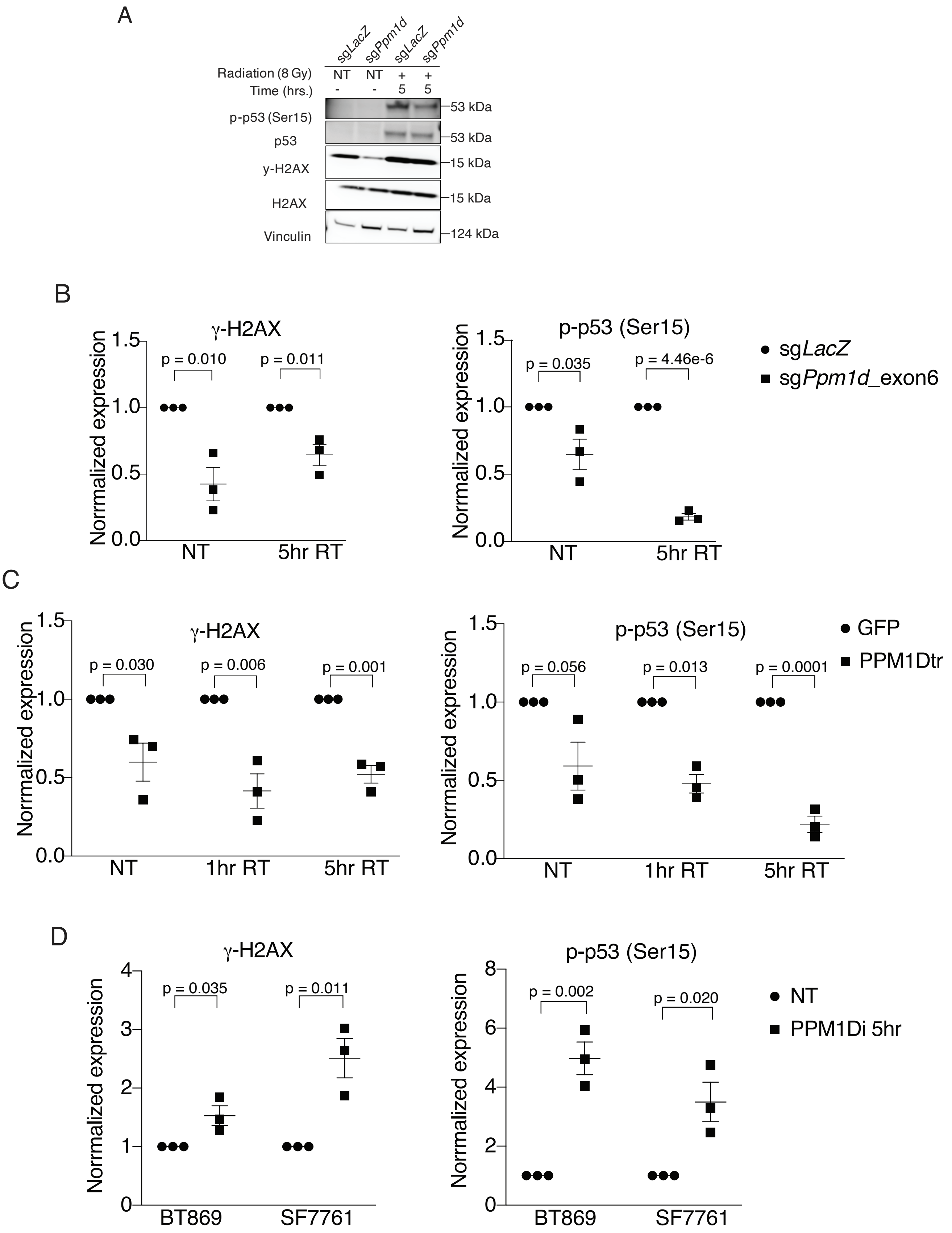

Supplementary Figure 6. A) mNSCs generated by endogenously truncating Ppm1d at exon 6 (sgPpm1d) or with non-targeting guides against LacZ (sgLacZ) were treated with 8 Gy of ionizing radiation (IR) and lysates were collected at baseline (NT) and 5 hours post-radiation respectively and probed with the indicated antibodies. B) Western blot quantification of protein expression levels of p-p53 (Ser15) and y-H2AX in IUE mNSC transfected with guides against LacZ (n=3) or Ppm1d exon 6 (n=3) at baseline as well as 5 hours post radiation treatment. Data presented as mean +/- S.E.M. from three independent experiments. P = 0.010 and 0.011 (y-H2AX) and 0.035 and 4.461e-6 (p-p53) for LacZ vs Ppm1d exon 6 at NT and 5hrs post radiation treatment calculated using two-tailed t-test. C) Western blot quantification of protein expression levels of p-p53 (Ser15) and y-H2AX in mNSC with H3F23A K27M overexpressing GFP (n=3) or PPM1Dtr (n=3) at baseline as well as 1 and 5 hours post radiation treatment. Data presented as mean +/- S.E.M. from three independent experiments. P = 0.030, 0.006, and 0.001 (y-H2AX) and 0.056, 0.013, and 0.0001 (p-p53) for GFP NT vs PPM1Dtr NT, GFP 1 hour vs PPM1Dtr 1 hour, and GFP 5 hours vs PPM1Dtr 5 hours conditions respectively calculated using two-tailed t-test. D) Western blot quantification of protein expression levels of p-p53 (Ser15) and y-H2AX in PPM1D-mutant DMGs BT869 (n=3) and SF7761 (n=3) at 5 hours after treatment with 10 uM of GSK2830371. Data presented as mean +/- S.E.M. from three independent experiments. P = 0.035 and 0.011 (y-H2AX) and 0.002 and 0.020 (p-p53) for BT869 and SF7761 cells respectively for NT vs PPM1Di 5hr condition calculated using two-tailed t-test. Source data are provided as a Source Data file.

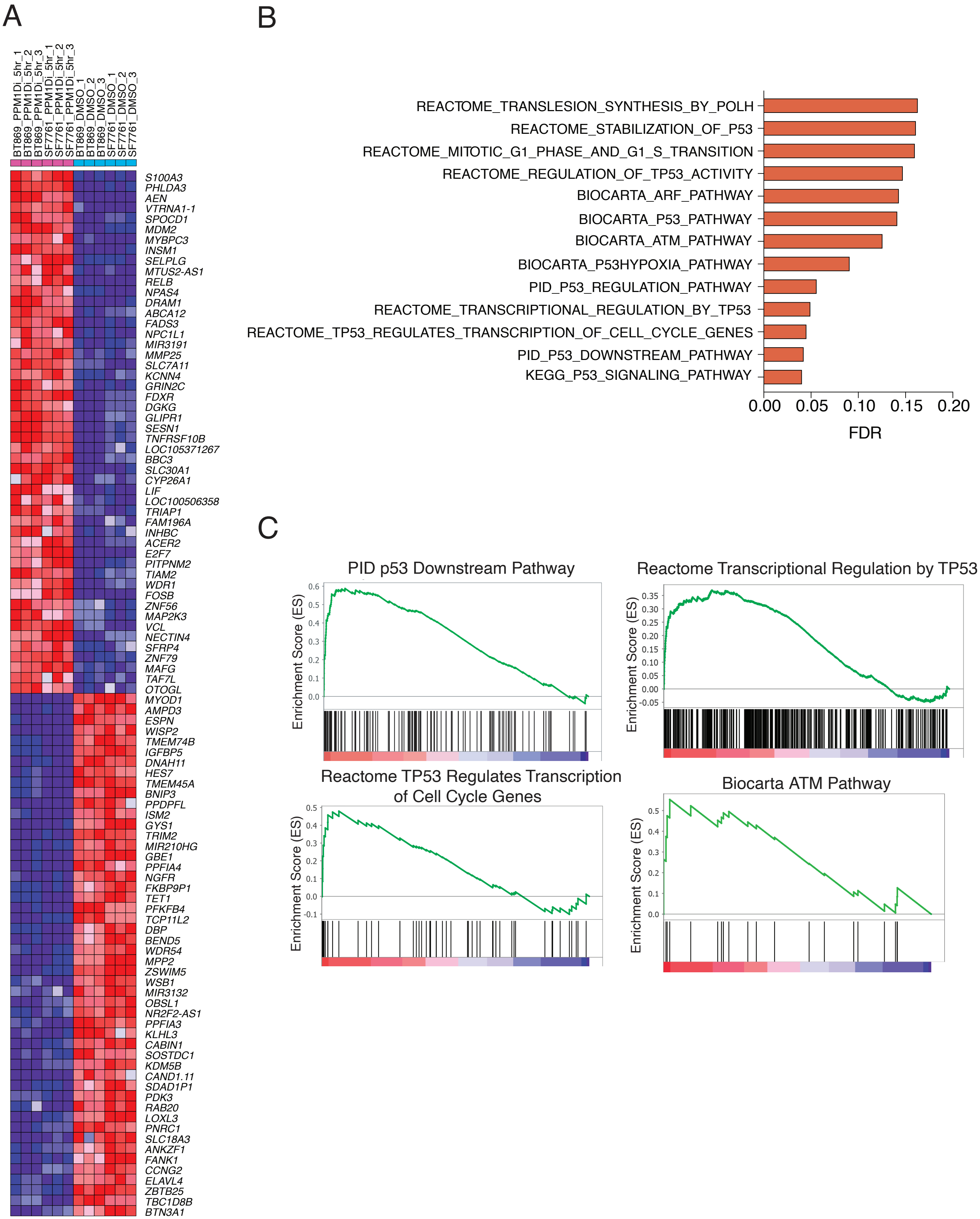

Supplementary Figure 7. A) Heatmap of top 100 differentially expressed genes between BT869 and SF7761 DMG cells (n=3 per cell line per condition) treated with 10 uM of GSK2830371compared to vehicle treated cells 5 hours post treatment. B-C) Pathways (B) and enrichment plots (C) related to p53, DDR and cell cycle that are significantly enriched (FDR < 0.25) after inhibition of PPM1D in BT869 and SF7761 cells.

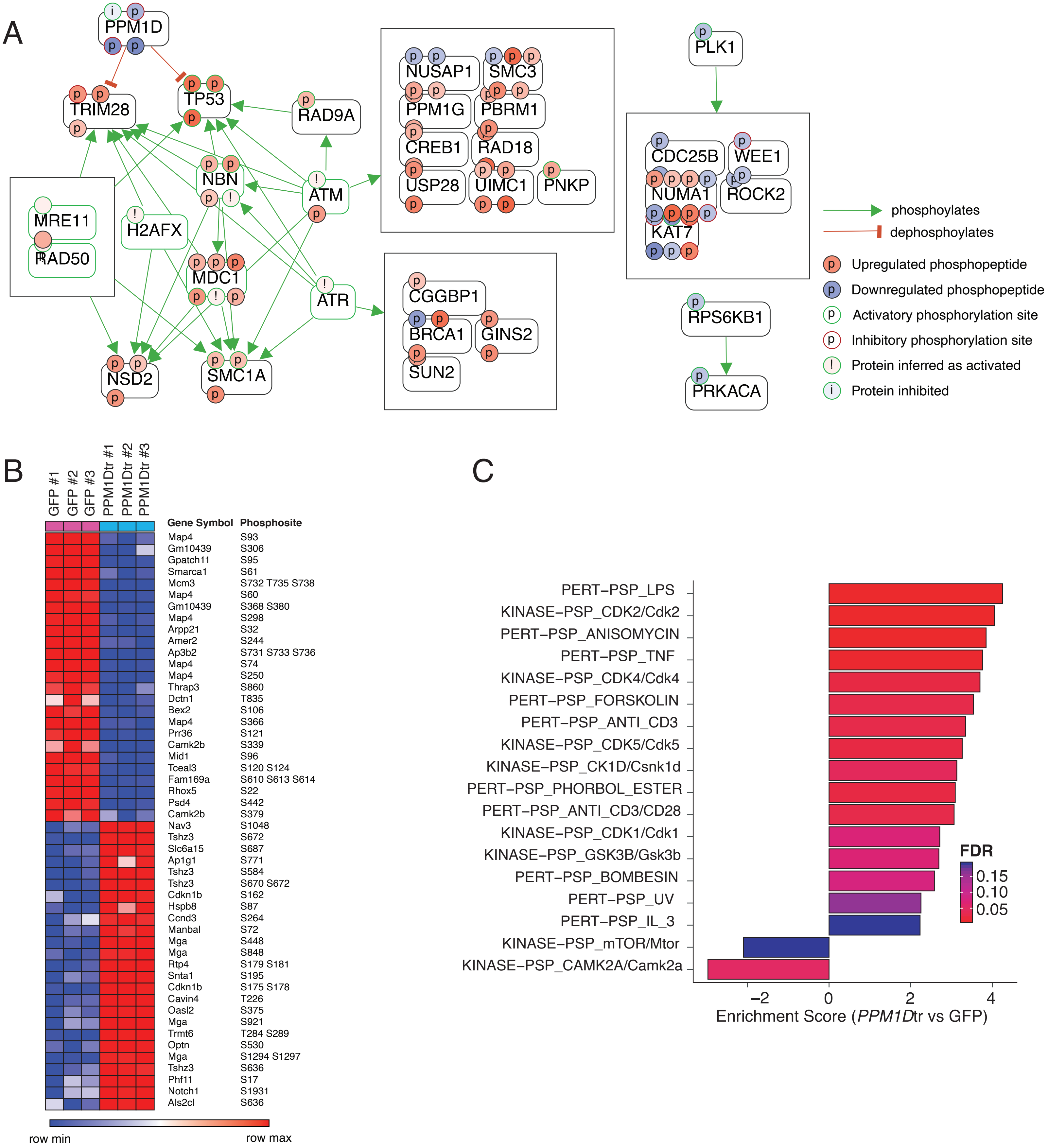

Supplementary Figure 8. A) CausalPath analysis of differentially altered phosphosites (FDR < 0.05) in BT869 cells between GSK2830371 and vehicle (DMSO) treated samples. Labels indicate whether the phosphosite is upregulated or downregulated, type of phosphorylation (activatory or inhibitory), and the prediction of whether a protein is activated or inhibited. B) Isogenic mNSCs overexpressing H3K3A K27M mutation plus either GFP (vector control) (n=3) or truncated PPM1D (PPM1Dtr) (n=3) were subjected to mass-spectrometry based phosphoproteomic analysis. Heatmap of top 50 differentially altered phosphosites (LFC > 1 and FDR < 0.01) between the two conditions are shown. C) Significantly enriched or downregulated pathways (FDR < 0.25) revealed by PTM-SEA analysis of the phosphosites between the two conditions. Positive and negative enrichment scores correspond to biological pathways upregulated and downregulated respectively in cells overexpressing H3F3A K27M plus PPM1Dtr compared to cells overexpressing H3F3A K27M plus GFP.

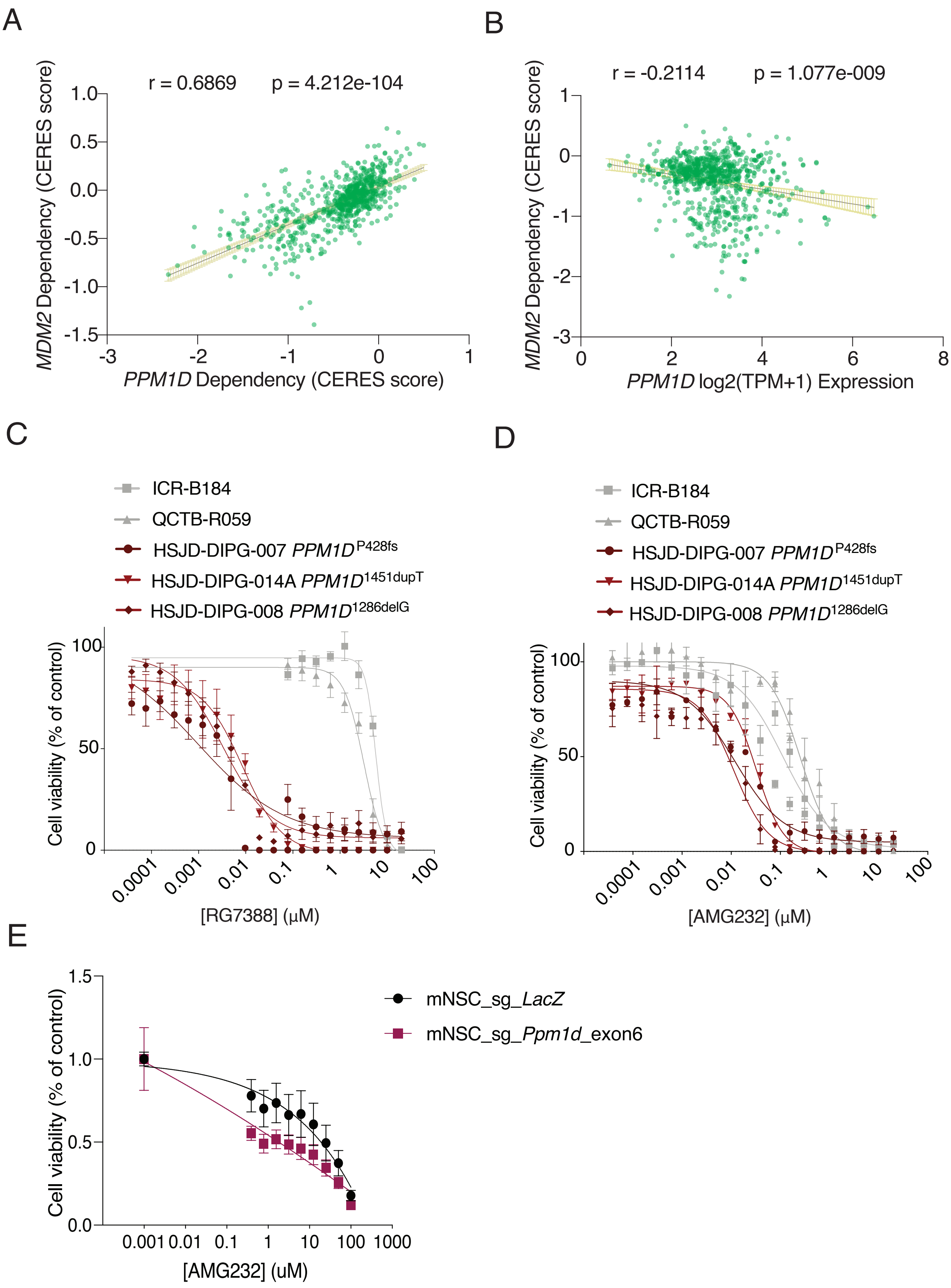

Supplementary Figure 9. A) Correlation of CERES dependency scores for PPM1D and MDM2 across 738 cancer cell lines. Pearson correlation and associated asymptotic p-value are shown. Error bars represent 95% confidence interval. B) Correlation of CERES dependency score for MDM2 and PPM1D Log2(TPM+1) expression across 816 cancer cell lines. Pearson correlation and associated asymptotic p-value are shown. Error bars represent 95% confidence interval. C-D) Drug response curves for a panel of three PPM1D-mutant and two PPM1D-WT DMG cell lines treated with different concentrations of MDM2 inhibitors RG7388 (C), and AMG232 (D) as indicated. E) Drug response curves for IUE mNSC transfected with guides against LacZ or Ppm1d exon 6 treated with indicated concentration of AMG232 MDM2 inhibitor. Data presented as mean +/- S.E.M. from three independent experiments. Source data are provided as a Source Data file.

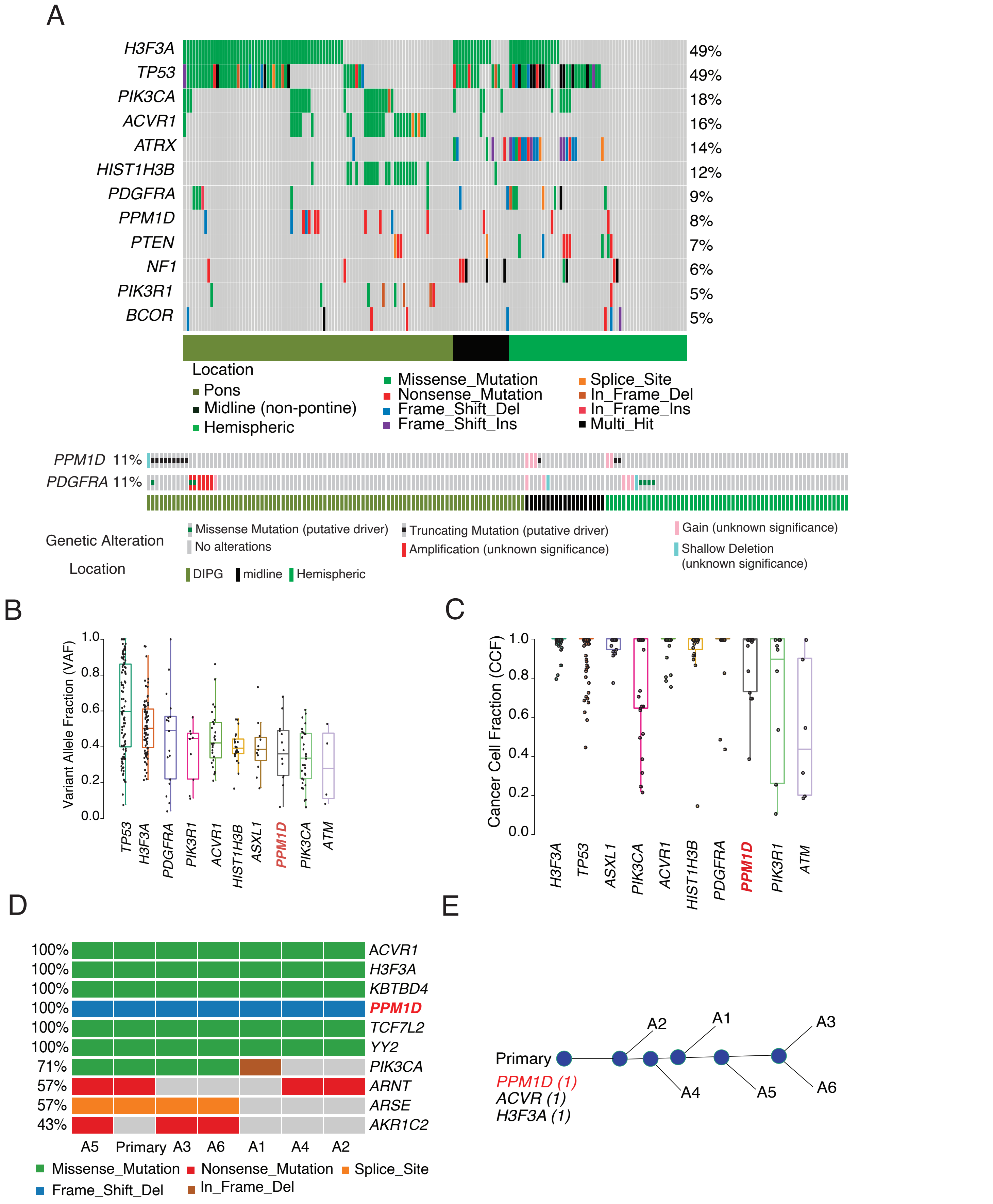

Supplementary Figure 10. A) Comutation plot showing all alterations depicted in Supplementary Figure 1A across 170 pediatric high-grade gliomas. Variants observed are depicted below. B) Variant allele fraction (VAF) of top recurrently altered genes in midline and non-midline gliomas (n=170). C) Cancer-cell fractions (CCF) of the top recurrently altered genes in pHGGs (n = 170). Bounds of the box represent the IQR, center represents the median, and the bounds of the whiskers represent 1.5 times IQR D-E) Comutation plot showing recurrent mutations in a primary DMG at diagnosis along with six multiregional samples of the same tumor obtained at autopsy (A1-A6) (D), and phylogenetic tree showing indicated truncal mutations (CCF = 1) in these samples (E). Blue circles depict presence of PPM1D/ACVR1/H3F3A mutations with CCF of 1 within each sample shown.

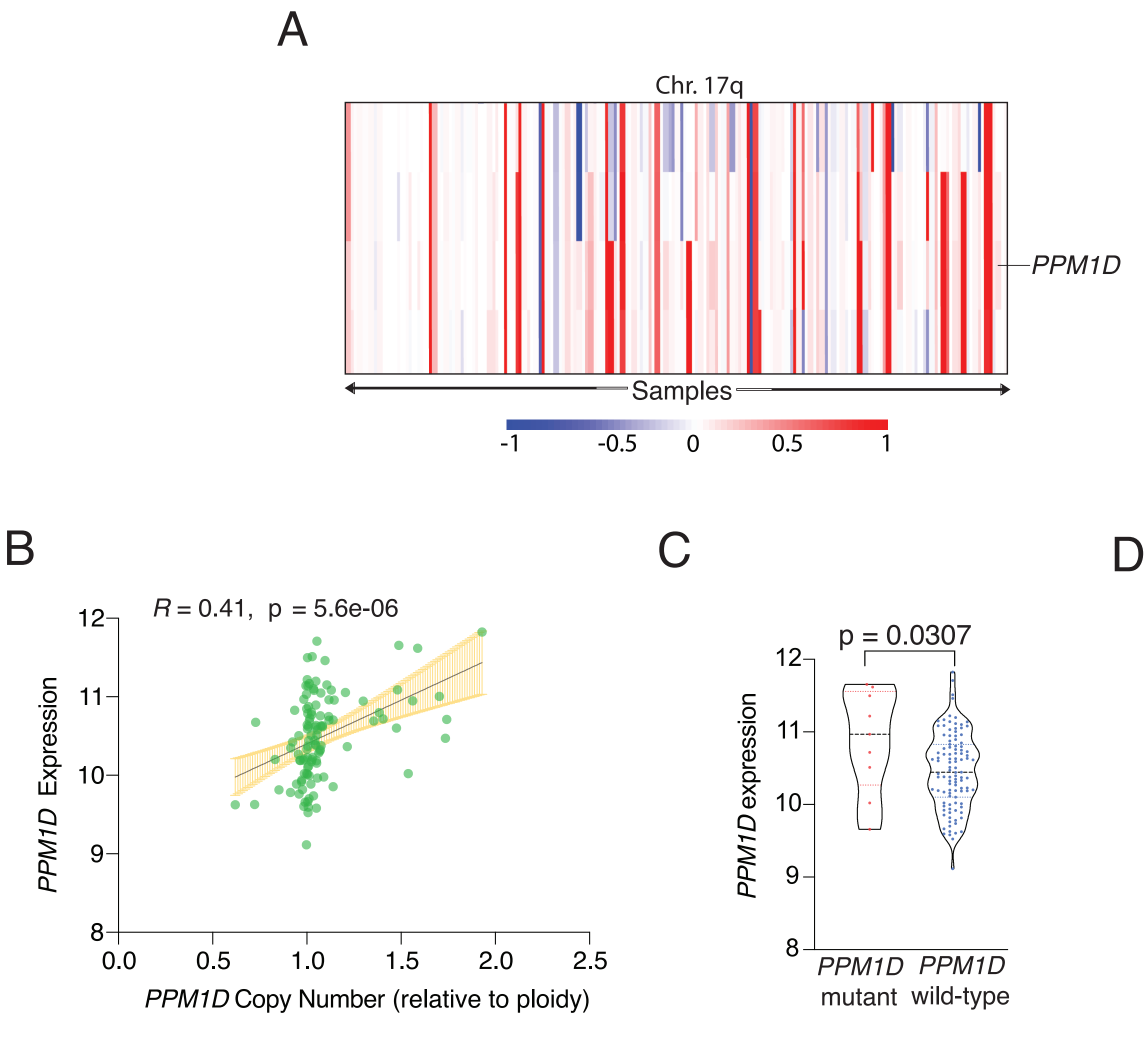

Supplementary Figure 11. A) Copy-number profile of chromosome 17q from our cohort of pHGG inferred using the GATK4 copy-number pipeline and GISTIC 2.0. Amplifications are shown in red and deletions are shown in blue. B) Correlation of PPM1D copy number and its expression across 112 human DMGs. Pearson correlation coefficient and associated two-tailed p-value of the coefficient are shown. Error bars represent 95% confidence interval. C-D) Violin plots comparing PPM1D expression levels between PPM1D-mutant (n=9) and wild-type (n=107) pHGG (C), or DMG (n=55) and other hemispheric tumors (n=58) (D). P = 0.0307 and 0.4270 for PPM1D-mutant vs wild-type and DMG vs hemispheric comparisons respectively calculated using two tailed t-test.
