# Supplementary material for: PPM1D mutations are oncogenic drivers of de novo diffuse midline glioma formation
Source: Nat Commun. 2022 Feb 1;13:604. doi: 10.1038/s41467-022-28198-8 (PMC8807747; doi:10.1038/s41467-022-28198-8)
Supplement: Supplementary file 3 — Description of Additional Supplementary Files [file 41467_2022_28198_MOESM3_ESM.pdf]

Description of Additional Supplementary Files

File Name: Supplementary Data 1
Description: Genes with expression Z-scores>2 in IUE DMG tumors with *Ppm1d* gRNA targeting exon 6, *PPM1D*-mutant human DMG, or in both sets. Related to Figure 2.

File Name: Supplementary Data 2
Description: Table containing mutational data on histones (*H3F3A* or *HIST1H3B*), *TP53* and *PPM1D* in various patient-derived cell lines used throughout the study.

File Name: Supplementary Data 3
Description: Differentially expressed genes (LFC>2, FDR<0.25) in the *PPM1D*-mutant cell lines BT869 and SF7761 comparing GSK2830371 treated cells to DMSO treated cells at 1 hour, 5 hours and 24 hours post treatment. Related to Figure 4.

File Name: Supplementary Data 4
Description: Gene-Set Enrichment Analysis (GSEA) analysis of the pathways significantly enriched (FDR <0.25) in the *PPM1D*-mutant cell lines BT869 and SF7761 comparing GSK2830371 treated cells to DMSO treated cells. Related to Figure 4.

File Name: Supplementary Data 5
Description: Phosphoproteomic analysis in *PPM1D*-mutant cell line BT869, comparing the GSK2830371 treated cells to DMSO treated cells. Related to Figure 5.

File Name: Supplementary Data 6
Description: STRING analysis of significantly associated biological pathways (FDR < 0.05) among proteins with top 50 significantly altered phosphosites (LFC > 1, FDR < 0.05) in BT869 cells, comparing the GSK2830371 treated cells to DMSO treated cells. Related to Figure 5.

File Name: Supplementary Data 7
Description: Phosphoproteomic analysis in mNSC, comparing *PPM1D* plus *H3F3A* K27M overexpressing mNSC to GFP plus *H3F3A* K27M overexpressing mNSC. Related to Supplementary Figure 7.

File Name: Supplementary Data 8
Description: Gene hits from genome-wide CRISPR screen in *PPM1D*tr overexpressing mNSC. Related to Figure 6.

File Name: Supplementary Data 9
Description: Gene-Set Enrichment Analysis (GSEA) of *PPM1D*tr-associated dependencies from the genome-wide CRISPR screen in *PPM1D*tr overexpressing mNSC. Related to Figure 6.

File Name: Supplementary Data 10
Description: Gene-Set Enrichment Analysis (GSEA) of the dependencies from the genome-wide CRISPR screen in wild-type (WT) mNSC. Related to Figure 6.

File Name: Supplementary Data 11
Description: Comparison of IC_{50} for several MDM2 inhibitors between the *PPM1D*-mutant and *PPM1D*-WT patient-derived cell lines. Related to Figure 6 and Supplementary Figure 8.

File Name: Supplementary Data 12
Description: Table showing cancer-cell fraction (CCF) of various genes in pre- and post-treatment samples in two different human DMGs. Related to Supplementary Note 1.
