# Supplementary material for: PPM1D mutations are oncogenic drivers of de novo diffuse midline glioma formation
Source: Nat Commun. 2022 Feb 1;13:604. doi: 10.1038/s41467-022-28198-8 (PMC8807747; doi:10.1038/s41467-022-28198-8)
Supplement: Supplementary file 16 — Reporting Summary [file 41467_2022_28198_MOESM16_ESM.pdf]

nature portfolio

Corresponding author(s): Pratiti Bandopadhayay and Timothy Phoenix

Last updated by author(s): Dec 7, 2021

Reporting Summary

Nature Portfolio wishes to improve the reproducibility of the work that we publish. This form provides structure for consistency and transparency in reporting. For further information on Nature Portfolio policies, see our Editorial Policies and the Editorial Policy Checklist.

Statistics

For all statistical analyses, confirm that the following items are present in the figure legend, table legend, main text, or Methods section.

n/a	Confirmed	
☐	☒	The exact sample size (n) for each experimental group/condition, given as a discrete number and unit of measurement
☐	☒	A statement on whether measurements were taken from distinct samples or whether the same sample was measured repeatedly
☐	☒	The statistical test(s) used AND whether they are one- or two-sided<br>*Only common tests should be described solely by name; describe more complex techniques in the Methods section.*
☒	☐	A description of all covariates tested
☐	☒	A description of any assumptions or corrections, such as tests of normality and adjustment for multiple comparisons
☐	☒	A full description of the statistical parameters including central tendency (e.g. means) or other basic estimates (e.g. regression coefficient) AND variation (e.g. standard deviation) or associated estimates of uncertainty (e.g. confidence intervals)
☐	☒	For null hypothesis testing, the test statistic (e.g. F, t, r) with confidence intervals, effect sizes, degrees of freedom and P value noted<br>*Give P values as exact values whenever suitable.*
☒	☐	For Bayesian analysis, information on the choice of priors and Markov chain Monte Carlo settings
☒	☐	For hierarchical and complex designs, identification of the appropriate level for tests and full reporting of outcomes
☐	☒	Estimates of effect sizes (e.g. Cohen's d, Pearson's r), indicating how they were calculated

Our web collection on statistics for biologists contains articles on many of the points above.

Software and code

Policy information about availability of computer code

Data collection	Flow cytometry data was acquired using BD LSR Fortessa instrument. Western blot images were acquired using ImageQuant LAS 4000. Data from proliferation and viability experiments performed with the Cell-Titer Glo luminescence method were collected using Molecular Devices SpectraMax M5. Data from live imaging proliferation experiments were collected using Essen BioScience IncuCyte S3. qRT-PCR data was collected using Applied Biosystems StepOnePlus Real-Time PCR system. Data were also accessed from publicly available portals such cBioPortal, TumorPortal and Tumorscape as described in the method section. The data from these portals were accessed on August 2019 for adult tumors and January 2021 for pediatric tumors.
Data analysis	Data analyses were performed using Microsoft Excel (version 16.54), Prism (9.0.1), and R-studio (1.4.1717). Flow cytometry data was analyzed using FlowJo (10.6.0). Bioinformatic pipelines and softwares used in the analysis of different genomic datasets are described in relevant parts of the method section.

For manuscripts utilizing custom algorithms or software that are central to the research but not yet described in published literature, software must be made available to editors and reviewers. We strongly encourage code deposition in a community repository (e.g. GitHub). See the Nature Portfolio guidelines for submitting code & software for further information.

Data

Policy information about availability of data

All manuscripts must include a data availability statement. This statement should provide the following information, where applicable:

- Accession codes, unique identifiers, or web links for publicly available datasets
- A description of any restrictions on data availability
- For clinical datasets or third party data, please ensure that the statement adheres to our policy

WGS along with corresponding RNA-sequencing data used in this study have been deposited to dbGaP under accession number phs002380.v1.p1 [https://www.ncbi.nlm.nih.gov/projects/gap/cgi-bin/study.cgi?study_id=phs002380.v1.p1]. RNA-seq data from mouse models and treatment of human DMG cell lines BT869 and SF7761 with GSK2830371 and DMSO have been deposited in GEO under accession number GSE179813 [https://www.ncbi.nlm.nih.gov/geo/query/acc.cgi?acc=GSE179813]. The proteomic data has been deposited to MassIVE [ftp://MSV000085700@massive.ucsd.edu]. Source data are provided with this paper.

Field-specific reporting

Please select the one below that is the best fit for your research. If you are not sure, read the appropriate sections before making your selection.

☒ Life sciences ☐ Behavioural & social sciences ☐ Ecological, evolutionary & environmental sciences

For a reference copy of the document with all sections, see nature.com/documents/nr-reporting-summary-flat.pdf

Life sciences study design

All studies must disclose on these points even when the disclosure is negative.

Sample size	All proliferation, qRT-PCR, and drug treatment assays were performed using at least three technical replicates. No sample size calculation was performed for in vitro experiments and the size was chosen based on prior experience. For in vivo studies, using approximately 10 mice per treatment group provided us with the ability to significantly (p=0.05) identify a 10-day difference in mean survival (18% difference in mean) with 80% power.
Data exclusions	Individual data from experiments performed using the IncuCyte live imaging system were excluded if the numbers differed significantly from other technical replicates and could be attributed to technical problems with scanning or cells sticking to the ULA 96 well plates.
Replication	All experiments were repeated with at least two or three biological replicates. All attempts at replication were successful.
Randomization	Not applicable to this study since our experiments did not require this.
Blinding	No blinding was performed in this study since our experiments did not require this.

Reporting for specific materials, systems and methods

We require information from authors about some types of materials, experimental systems and methods used in many studies. Here, indicate whether each material, system or method listed is relevant to your study. If you are not sure if a list item applies to your research, read the appropriate section before selecting a response.

Materials & experimental systems

n/a	Involved in the study	
☐	☒	Antibodies
☐	☒	Eukaryotic cell lines
☒	☐	Palaeontology and archaeology
☐	☒	Animals and other organisms
☐	☒	Human research participants
☒	☐	Clinical data
☒	☐	Dual use research of concern

Methods

n/a	Involved in the study	
☒	☐	ChIP-seq
☐	☒	Flow cytometry
☒	☐	MRI-based neuroimaging

Antibodies

Antibodies used	Following antibodies were commercially purchased from indicated vendors:<br>Anti-phospho-p53 (Ser15) antibody (D4S1H); Cell Signaling Technology Cat# 12571<br>Anti-p53 antibody (1C12); Cell Signaling Technology Cat# 2524<br>Anti-phospho-Histone (H2A.X) (Ser139) antibody (20E3); Cell Signaling Technology Cat# 9718<br>Anti-Histone H2A.X antibody; Cell Signaling Technology Cat# 2595<br>Anti-p53 antibody (DO-1); Santa Cruz Biotechnology Cat# sc126

	Anti-Vinculin antibody; Sigma Aldrich Cat# V9131<br>Anti-V5 antibody; Thermo Fisher Scientific Cat# R960-25<br>Anti-Phopho-p53 (Ser15) antibody; Cell Signaling Technology Cat# 9284<br>Anti-mouse IgG, HRP-linked antibody; Cell Signaling Technology Cat# 7076<br>Anti-rabbit IgG, HRP-linked antibody; Cell Signaling Technology Cat# 7074<br>Anti-Olig2 antibody; Millipore Cat# Ab9610<br>Anti-Gfap antibody; Cell Signaling Technology Cat# 12389<br>Anti-Ki67 antibody; Cell Signaling Technology Cat# 9129<br>Anti-GFP antibody; Thermo Fisher Scientific Cat# A11122
Validation	All antibodies were validated by the manufacturers indicated above. The validation of these antibodies can found in the respective vendor websites.

Eukaryotic cell lines

Policy information about cell lines

Cell line source(s)	SU-DIPG-IV, SU-DIPG-XIII, SU-DIPG-XVII were obtained from Dr. Michelle Monje at Stanford University, and BT869 cell lines were obtained from the DFCI Center for Patient Derived Models (CPDM). HCT116 and HEK-293T cells were obtained from Broad Institute's Cancer Cell Line Factory (CCLF). SF7761 cell line was purchased from Sigma Aldrich (SCC126). HSJD-DIPG-007, HSJD-DIPG-008, HSJD-DIPG-014A, ICR-B184, and QCTB-R059 cells were obtained from Drs. Chris Jones and Angel Carcaboso.
Authentication	All patient derived DMG cell lines were authenticated using SNP-based fingerprinting and/or targeted Sanger sequencing.
Mycoplasma contamination	All cells used were tested negative for mycoplasma using the MycoAlert Mycoplasma Detection Kit.
Commonly misidentified lines<br>(See ICLAC register)	No commonly misidentified cells were used in the study.

Animals and other organisms

Policy information about studies involving animals; ARRIVE guidelines recommended for reporting animal research

Laboratory animals	Mice: CD1-IGS strain (Charles river code 022). IUE was performed on e13.5-14.5 timed pregnant females. IUE offspring included both male and female for all glioma mouse models.
Wild animals	There were no wild animals used in this study.
Field-collected samples	There were no field-collected samples used in this study.
Ethics oversight	All mouse work was done according to institutional and IACUC review boards (University of Cincinnati).

Note that full information on the approval of the study protocol must also be provided in the manuscript.

Human research participants

Policy information about studies involving human research participants

Population characteristics	The WGS dataset consisted of 131 pre-treatment pHGGs (76 DMGs and 55 non-midline pHGGs) and 39 post-treatment pHGGs (34 DMGs and 5 non-midline pHGGs). Among patients with available demographic information, there were equal proportions of males and females and the age ranged from 0.1 years to 31 years with mean age of 9.49 years.
Recruitment	Tumor and matched normal samples from patients with pediatric high-grade gliomas (pHGG), including DMG, were collected at the Dana-Farber Cancer Institute and collaborating institutions. Additional WGS data analyzed were downloaded from previously published studies (Buczkowicz et al., 2014; International Cancer Genome Consortium PedBrain Tumor, 2016; Taylor et al., 2014; Wu et al., 2014). Detailed information about recruitment can be found in the Dubois et al. (unpublished) study.
Ethics oversight	Ethics approval was granted by the relevant human IRB of Dana-Farber Cancer Institute (DFCI) and collaborating institutions. All patients provided informed consent prior to collection of samples or were analyzed as de-identified samples with specific IRB waiver of informed consent. Detailed information about ethics oversight can be found in the Dubois et al. (unpublished) study.

Note that full information on the approval of the study protocol must also be provided in the manuscript.

Flow Cytometry

Plots

Confirm that:

☒ The axis labels state the marker and fluorochrome used (e.g. CD4-FITC).

☒ The axis scales are clearly visible. Include numbers along axes only for bottom left plot of group (a 'group' is an analysis of identical markers).

☒ All plots are contour plots with outliers or pseudocolor plots.

☒ A numerical value for number of cells or percentage (with statistics) is provided.

Methodology

Sample preparation	Annexin V-APC (Thermo Fisher, A35110) and Propidium iodide (Thermo Fisher, NC9699940) were used to determine the proportion of early and late apoptotic cells as previously described (Bandopadhayay et al., 2014). Proportion of cells in different phases of the cell cycle was determined by flow cytometric assessment using the APC BRDU/7-AAD Flow Kit (BD biosciences, 556454) as per manufacturer's instructions.
Instrument	BD LSR Fortessa
Software	FlowJo
Cell population abundance	At least 10,000 cells were analyzed in all apoptosis and cell cycle experiments.
Gating strategy	Cells were discriminated from debris and clumps using the FSC-A/SCC-A gating strategy based on experience. Only single cells were used using FSC-H/FSC-A gating strategy and selecting cells along the diagonal. For apoptosis experiments, cells were designated as necrotic, late apoptotic, early apoptotic, or live based on their relative Annexin V and PI staining levels. For cell cycle experiments, cells were designated as G0-phase, S-phase, or G2/M phase based on their relative 7-AAD and BrdU staining levels.

☒ Tick this box to confirm that a figure exemplifying the gating strategy is provided in the Supplementary Information.
